# Extraction of phenotypic traits from taxonomic descriptions for the tree of life using natural language processing

**DOI:** 10.1002/aps3.1035

**Published:** 2018-03-31

**Authors:** Lorena Endara, Hong Cui, J. Gordon Burleigh

**Affiliations:** ^1^ Department of Biology University of Florida Gainesville Florida 32611 USA; ^2^ School of Information University of Arizona Tucson Arizona 85719 USA

**Keywords:** morphological matrices, natural language processing, phenotypic traits, taxonomic descriptions

## Abstract

**Premise of the Study:**

Phenotypic data sets are necessary to elucidate the genealogy of life, but assembling phenotypic data for taxa across the tree of life can be technically challenging and prohibitively time consuming. We describe a semi‐automated protocol to facilitate and expedite the assembly of phenotypic character matrices of plants from formal taxonomic descriptions. This pipeline uses new natural language processing (NLP) techniques and a glossary of over 9000 botanical terms.

**Methods and Results:**

Our protocol includes the Explorer of Taxon Concepts (ETC), an online application that assembles taxon‐by‐character matrices from taxonomic descriptions, and MatrixConverter, a Java application that enables users to evaluate and discretize the characters extracted by ETC. We demonstrate this protocol using descriptions from Araucariaceae.

**Conclusions:**

The NLP pipeline unlocks the phenotypic data found in taxonomic descriptions and makes them usable for evolutionary analyses.

Understanding the evolution of phenotypic traits is critical for resolving the genealogy of life and elucidating interactions of organisms with their environments through time. Although phenotypic data are necessary to address many evolutionary questions, assembling phenotypic data from diverse taxa across the tree of life can be extremely labor intensive and costly.Several initiatives have developed tools to expedite the assembly of phenotypic data sets (Furbank and Tester, 2011). New high‐throughput phenotyping approaches and image analysis tools can automate the acquisition of quantitative traits from two‐ or three‐dimensional images (Hartmann et al., 2011; Viscosi and Cardini, 2011; Fahlgren et al., 2015; Rahaman et al., 2015; Gehan and Kellogg, 2017; Lelievre and Grey, 2017). Furthermore, new crowdsourcing tools enable large groups of non‐experts to score traits from images of diverse taxa (e.g., O'Leary et al., 2018). However, simply obtaining appropriate and useful images for many taxa can be difficult, and these methods may be limited to a small set of pre‐defined characters. Taxonomic descriptions often describe a broader range of character traits, including both qualitative and quantitative traits that provide a summary of the variation observed within a taxon (e.g., length of leaf: 6–10 cm; shape of leaf: ovate to obovate). Consequently, recent research has focused on developing the infrastructure, including software, glossaries, and ontologies, to automate the large‐scale extraction of phenotypic data from taxonomic descriptions (Jaiswal et al., 2005; Cui, 2012; Burleigh et al., 2013; Hamman et al., 2014; Garnier et al., 2016; Hoendorf et al., 2016; Endara et al., 2017).We describe a natural language processing (NLP) pipeline that leverages this new infrastructure to build character‐by‐taxon phenotypic trait matrices that are usable for evolutionary inference from formal taxonomic descriptions written in English. The NLP pipeline uses a non‐supervised learning strategy that analyzes the full length of the body of a description. Therefore, it can be used for character discovery of both qualitative and quantitative characters (Cui, 2012). Although the NLP pipeline can extract phenotypic data sets from different groups of organisms besides plants (e.g., Daly et al., 2015; Cui et al., 2016), it includes a built‐in glossary of over 9000 botanical terms (Endara et al., 2017), which makes it especially well suited for assembling plant trait matrices. We demonstrate how the NLP pipeline can quickly jump‐start the assembly of a phenotypic character matrix using the gymnosperm family Araucariaceae as an example.

## METHODS AND RESULTS

**Figure 1:**
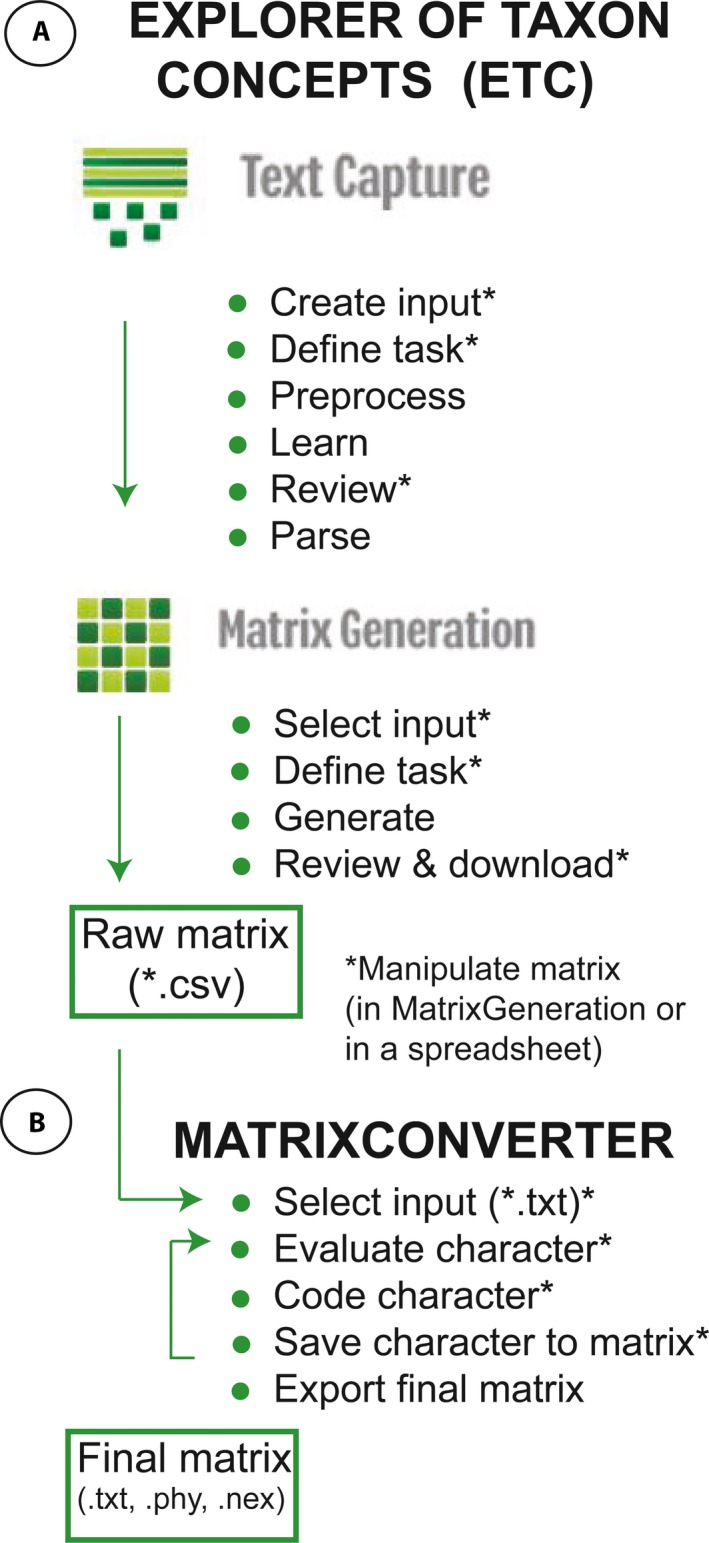
Software and steps of the natural language processing pipeline used to extract phenotypic traits from taxonomic descriptions. (A) Explorer of Taxon Concepts, (B) MatrixConverter. * indicates steps where human input is required.

**Figure 2:**
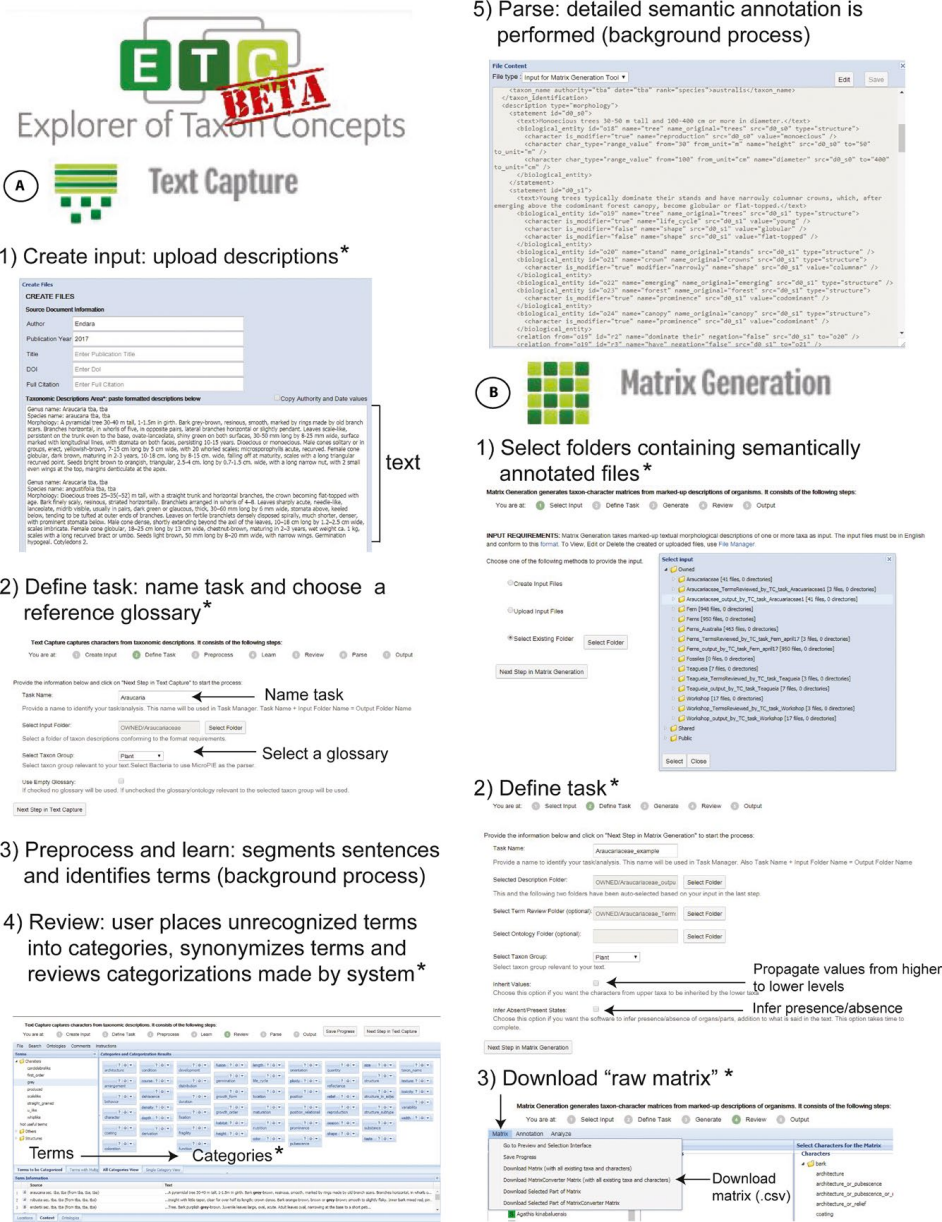
Explorer of Taxon Concepts tools and steps used to extract taxonomic information from taxonomic descriptions and generate a phenotypic matrix. (A) Text Capture tool, (B) Matrix Generation tool. * indicates steps where human input is required.

The NLP pipeline used to parse and extract phenotypic characters from taxonomic descriptions includes the Explorer of Taxon Concepts (ETC; Cui et al., 2016) and MatrixConverter (Liu et al., 2015). ETC (Figs. 1A, 2) is an online application (http://etc.cs.umb.edu/etcsite/) that contains the Text Capture and Matrix Generation tools, which are used to parse text and assemble a character matrix. MatrixConverter (Fig. 1B, Appendix 1) is a Java application (available on GitHub at https://github.com/gburleigh/MatrixConverter/tree/master/distribution) that facilitates the evaluation and discretization of the characters extracted by ETC and the formatting of the resulting character matrices.

The initial input for the ETC's Text Capture Tool consists of text contained in the body of taxonomic descriptions written in English using a telegraphic syntax. In botanical descriptive literature, the most common telegraphic syntax format is characterized by its abbreviated format that drops auxiliary verbs and unnecessary terms (Fig. 2A, step 1). Taxonomic descriptions document a taxon's phenotypic traits and its variation; thus, the traits extracted represent summary information of a taxon and not of an individual. Ideally, users should select descriptions that represent the most up‐to‐date or credible circumscription of taxa. Furthermore, using descriptions written for the same taxonomic treatment (e.g., floras, monographs) by one or a few authors during the same time period may facilitate the analysis and result in a more complete matrix, as they are more likely to be parallel and have a more consistent use of language. Extended sections of descriptions, which often include descriptions of habitat and discussion of diagnostic characters, should not be included in the analysis because they are written in natural (complete) language and will not work well with the ETC parser. Parenthetical remarks that explain a trait or compare it to other taxa (e.g., … distal cells quadrate [including rhombic] to hexagonal; …in cross‐section [mid‐limb], …cone [larger than other species of the genus] 10 cm long…) should also be excluded from the analysis because they often violate the rules of telegraphic syntax and hinder the ETC parsing analysis.Users first upload properly formatted descriptions, including the taxon name and descriptive text, into the ETC via the File Manager or Text Capture tool; they can upload a single description or multiple descriptions using the batch upload option (Fig. 2A, step 1; ETC offers examples of the format for each option). Users can include documentation of the bibliographic source of the description and information of the author and year of the description. Including taxonomic descriptions that represent various taxonomic hierarchical levels (e.g., generic, species) may reduce the amount of missing data in the resulting character matrix, as higher‐level descriptions may describe traits that are not mentioned in lower‐level descriptions. ETC makes it possible for the lower‐level taxa to inherit characters from higher‐level taxa. After ETC validates the input descriptions, the user assigns a name to their task and selects a reference glossary. For analyses of plant groups, the user can select the built‐in Plant Glossary (Fig. 2A, step 2). The Plant Glossary (OTO Glossary version 0.19) is a controlled vocabulary specifically assembled to parse botanical taxonomic literature (Endara et al., 2017). Currently it consists of 9228 terms extracted from the 30 volumes of the floras of North America and China (Flora of North America Editorial Committee, 1993+; Flora of China Editorial Committee, 1994+), which are grouped into 53 categories. Although using the Plant Glossary will expedite the extraction of plant traits, ETC makes it possible to build or input alternate glossaries. To begin building a glossary, the user can check “Use empty glossary.”Once the descriptions are uploaded, the software transforms the text of the descriptions into extensible markup language (XML) format and subsequently segments the sentences and analyzes the text using a non‐supervised learning strategy (Cui, 2012; Fig. 2A, step 3). In this step, the software uses the glossary to learn phenotypic terms in the descriptions. A term is said to be “learned” by the software if the software can classify the new term to either the structure or character categories. The software can still learn many terms from the text directly without a glossary. Both types of terms, terms that are recognized or learned by the system (i.e., exist in the reference plant glossary) and terms that are unrecognized by the software, are uploaded into the next phase of the analysis (Fig. 2A, step 4) for the user to review. The ETC term review step provides a user‐friendly interface that enables the user to refine the categorization of terms by dragging and dropping unrecognized terms into predefined categories (Fig. 2A, step 4). The software uses the terms and the corresponding categories to build an “is_a” ontological relationship that helps to annotate the text (i.e., ash‐gray is_a Coloration, u‐like is_a Shape). To control the different ways in which humans express the same quality or structure, the software also allows the user to define synonyms (e.g., shiny = glossy), or place the same term into multiple categories (e.g., “scale” placed in the Structure category; “scale” placed in the Shape category), and share the task of categorizing terms with other users. The next “parsing” step is a background process during which the software parses and semantically annotates the text (Fig. 2A, step 5). The output of this step consists of (1) a series of detailed semantically annotated XML files, with one file representing each taxon provided in the input, and (2) a categorical glossary resulting from the user's categorization of the terms.The XML output files of the Text Capture tool are input in the ETC Matrix Generation tool (Fig. 2B, step 1), which assembles a taxon‐by‐character matrix. When defining the task (Fig. 2B, step 2), the Matrix Generation tool provides the option of propagating traits extracted from higher‐level taxonomic descriptions (e.g., from genus descriptions) into the corresponding cells of the lower‐level taxa. Furthermore, it provides the option of inferring the presence of structures mentioned in descriptions. For example, if “petiole red” is described for taxon A, this option will infer that the petiole is present in taxon A; if the “petiole” is not mentioned in taxon B, it will be shown in the matrix as missing information, and if desired, the user can manually populate the absence values. Although these options can help quickly populate the resulting character matrix, they should be used cautiously. The matrix output by ETC (called the “raw matrix” hereafter) includes the exact phrases extracted from the descriptions, which may need to be transformed or edited before analyses. Users can merge characters or correct the spelling or formatting in the raw matrix using the ETC Matrix Generation tool, but for large data sets we recommend downloading the raw matrix and modifying it using a spreadsheet, as large matrices can overload the browser. Users can download the raw matrix immediately after the Matrix Generation step is completed or through the Matrix tab → Download Matrix (with all existing taxa and characters) command (Fig. 2B, step 3).Assessing the validity and homology of the characters and character states, as well as delimiting, discretizing, and coding the characters of the raw matrix, is critical for evolutionary inference using phenotypic characters. MatrixConverter (Appendix 1) provides a user‐friendly interface designed to facilitate the evaluation and discretization of qualitative and quantitative phenotypic characters (Appendices 1A and B, respectively). After the user selects the useful characters and codes them, MatrixConverter can export the final matrix in different formats (e.g., PHYLogeny Inference Package [PHYLIP], Nexus, text, NeXML) that can be used in many evolutionary inference programs.To demonstrate the ability of the NLP pipeline to extract phenotypic data from taxonomic descriptions that could be used in phylogenetic inference, we input 41 taxonomic descriptions, including two generic and 39 species‐level descriptions (Table 1), of the gymnosperm family Araucariaceae into the ETC Text Capture tool (Input Generator Tool version 1.0, Semantic Markup version 0.1.195‐SNAPSHOT). The preprocessing and learning phases of the analysis took 3 min 15 s. The time it takes to complete the next step—classifying unknown terms and checking the classification decisions made by the software—can vary greatly depending on the number of terms and the user's familiarity with the software and with the technical vocabulary used in the descriptions. In our analysis, it took 21 min 44 s to classify 84 unrecognized terms, identify and establish equivalence (i.e., synonymy) among terms, and verify 441 terms pre‐classified by the system based on the Plant Glossary. Finally, it took 13 min 27 s to parse and semantically annotate the text and 2 min 2 s to assemble the matrix using the Matrix Generator tool (version 0.1.56‐SNAPSHOT). We used the default options of Matrix Generator that did not propagate values from higher‐level descriptions or infer presence/absence, as we decided to perform this task manually to ensure accuracy. The total time of the parsing analysis and matrix generation was 37 min 30 s. The resulting raw matrix consisted of 509 characters, of which 33 were found only in higher‐level descriptions (i.e., generic descriptions). Among the characters in the matrix, 83% had data from fewer than four taxa, and overall the matrix was 7.1% filled.

**Table 1:** Taxa of the Araucariaceae included in the natural language processing analysis

Genus^a^	No. of characters in raw matrix^b^	No. of characters in final matrix
***Agathis*** 1	61	
*A. atropurpurea* 2	27	26
*A. australis* 2	52	33
*A. borneensis* 2	62	27
*A. corbassonii* 2	31	33
*A. dammara* 2	58	36
*A. endertii* 2	29	27
*A. flavescens* 2	30	30
*A. kinabaluensis* 2	29	31
*A. labillardieri* 2	31	31
*A. lanceolata* 2	38	33
*A. lenticula* 2	29	34
*A. macrophylla* 2	55	31
*A. microstachya* 2	51	30
*A. montana* 2	29	31
*A. moorei* 2	37	33
*A. orbicula* 2	26	29
*A. ovata* 2	39	34
*A. robusta* 2	53	31
*A. silbae* 2	53	41
***Araucaria*** 1	32	
*A. angustifolia* 2	40	33
*A. araucana* 2	43	34
*A. bernieri* 2	34	37
*A. bidwillii* 2	56	27
*A. biramulata* 2	33	35
*A. columnaris* 2	31	35
*A. cunninghamii* 2	32	35
*A. heterophylla* 2	27	35
*A. humboldtensis* 2	26	36
*A. hunsteinii* 2	24	32
*A. laubenfelsii* 2	35	39
*A. luxurians* 2	28	37
*A. montana* 2	27	40
*A. muelleri* 2	31	38
*A. nemorosa* 2	29	34
*A. rulei* 2	38	39
*A. schmidii* 2	20	28
*A. scopulorum* 2	36	36
*A. subulata* 2	25	32
*Wollemia nobilis* 3	27	32

^a^Superscript numbers indicate the source of the taxonomic description: ^1^Farjon, 2010; ^2^Earle, 2006; ^3^Jones et al., 1995.

^b^Prior to the inclusion of characters extracted from higher‐level descriptions.

Although generating a raw matrix takes only minutes, regardless of the data set, evaluating and coding characters can be a time‐consuming task that is difficult to automate. Due to the size of the Araucariaceae matrix, we downloaded it and identified characters that needed to be merged using a spreadsheet (Microsoft Excel; Microsoft Corporation, Redmond, Washington, USA). This is necessary because authors of descriptions use different expressions to refer to the same structure (e.g., “bracts,” “cone bracts,” “cone‐bract”), and if the user does not synonymize these expressions during the Term Review step, the ETC software identifies these as different structures and generates characters for each of them. Synonymies can be difficult to detect. Therefore, users should carefully evaluate all the contexts of a term before categorizing the term or establishing synonymies (Endara et al., 2017). While manipulating the raw matrix, we manually added selected characters extracted from the generic descriptions. Once manipulation of the raw matrix was complete, we evaluated the 84 characters with data from four or more taxa. It took a single user (L. Endara, a non‐expert in the Araucariaceae) 13 h 42 min to evaluate and code the characters for the final matrix. The final Araucariaceae matrix consisted of 71 characters (Appendix 2, Appendix S1), seven of which were extracted from the generic descriptions and added manually; the final matrix was 47% complete. The matrix included 54 qualitative and 17 quantitative characters (Appendix 2, Appendix S1). Many of the characters in the final matrix described shapes (23%) and colors (8%) of structures. The structures with the most characters were leaves (15 characters), followed by the female and male cones (13 and 11 characters, respectively). We compared the phenotypic characters in the final matrix to those used in a morphological matrix of Araucariaceae from Escapa and Catalano (2013). We found that 31% of the characters of both data sets overlap (Appendix 2). However, our final matrix lacked characters associated with micromorphology and anatomical features that were not included in descriptions (e.g., stomata, subsidiary cells, detailed vascularization patterns), as well as characters that summarize two or more structures or traits (e.g., ovuliferous complex encompassing the bract and scale, ratios bract/scale length), but our approach extracted phylogenetically informative characters for structures not considered by Escapa and Catalano (2013) (e.g., characters associated with the bark and branching pattern).

## CONCLUSIONS

The ETC tool, which includes a built‐in reference glossary specifically created to parse a technical botanical vocabulary, enables the extraction of plant phenotypic traits from the legacy taxonomic and natural history literature. The ETC collaborative environment allows users to share their tasks with other users, a feature that enables the participation of users with different levels of expertise who can contribute to different phases of the analysis. Within ETC, the MatrixConverter NLP pipeline provides an efficient, semi‐automated approach to extract phenotypic traits from large numbers of taxonomic descriptions at different hierarchical levels. The power of the NLP approach relies on its speed and ability to handle the linguistic complexity of the text written for diverse taxonomic groups by different authors. Although we present an example using 31 taxonomic species of three genera of the Araucariaceae, we also have used this pipeline to parse 950 descriptions of the pteridophyte flora of Mexico (Mickel and Smith, 2004) and 722 descriptions of conifers (Earle, 2006). Users should be aware that with more taxonomic descriptions, there is a higher likelihood that terms will be used in an inconsistent manner, and this may result in larger raw matrices that require extensive editing.In our experience with the NLP pipeline, many of the characters have few data points (i.e., data from few taxa), resulting in matrices that have a high proportion of missing data. In our Araucariaceae example, the raw matrix had data in only 7.1% of the cells, whereas the final matrix had data in 47% of the cells. This high proportion of missing data is common across data sets and can be partly attributed to the authors’ tendency to emphasize the diagnostic and/or unique features of a taxon over more common, shared features that may not be included in the descriptions. Using parallel descriptions in the NLP pipeline will likely produce more complete matrices. Nevertheless, as demonstrated by our example, this method extracts a significant amount of useful data even when using non‐optimal descriptions (i.e., different description sources and authors indicated in Table 1). Although the resulting matrix (i.e., raw matrix) may not represent the final, complete matrix for evolutionary inference, the NLP pipeline provides a fast jump‐start for building a large phenotypic trait matrix that can be used in a variety of disciplines (e.g., functional traits and community assembly; see Sessa et al., 2018).Every data set presents unique challenges for the NLP pipeline, as the use of terms often varies both within and between sets of taxonomic descriptions. For example, in the Araucariaceae data set, “cone‐scales” and “scales” were used to refer to the same structure, but “scales” is also used to describe the shape and type of leaves (e.g., adult leaves scale‐like) and a pattern of exfoliation (e.g., bark exfoliating in fine scales). Furthermore, terms and expressions used in one group might not be equivalent in other groups. For example, “scales” is also used to describe the relief of surfaces in other (i.e., non‐gymnosperm) groups; in the fern genera *Pleopeltis* Humb. & Bonpl. ex Willd. and *Haplopteris* C. Presl peltate scales cover the immature sori and the rhizome scales are dark brown, respectively, and in *Eriophorum* L. of the Cyperaceae, the spikelets have scales that are spirally arranged. Before classifying the terms (Fig. 2A, step 4), users should not assume the equivalence of terms unless they carefully evaluate the term in its context within the description.Cui et al. (2016) addressed the quality and accuracy of the quantitative characters extracted from spider descriptions using this NLP pipeline, compared the resulting matrices against a gold standard matrix, and found a precision/recall of 99.79%/98.92%. Based on these findings and subsequent analyses, Cui and collaborators further optimized the ETC pipeline and offered suggestions for best practices for authors of descriptions. Here we demonstrate that the pipeline can also efficiently extract qualitative plant traits for use in evolutionary analyses. Processing and analyzing new collections of taxonomic descriptions from across the tree of life with the NLP pipeline leads us to discover new expressions and grammatical constructions that help to optimize the NLP pipeline and its components, like the Plant Glossary. In the future, the ETC pipeline will allow users to import and create ontologies, hierarchical organizations of terms that establish relationships among structures, entities, and qualities and enable the computer to have reasoning capabilities (Dececchi et al., 2015). Using ontologies will likely increase the number of usable phenotypic characters obtained by the NLP pipeline. For example, currently some substructures like “base” cannot be related to their parent structure, such as “tree” or “leaf.” Therefore, the user may not be able to discern if the information the ETC pipeline extracted under the “size of the base” describes the “base of the plant” or “base of the leaf.” By incorporating ontologies in the NLP pipeline, the software can create a bridge between “base” and its parent structure (i.e., plant or leaf). In addition to extracting phenotypic characters that can be used in the exploration of the plant tree of life, the terms extracted and traits generated using this pipeline will help expand other infrastructures that seek to make terms comparable, inferable, and searchable (e.g., Plant Ontology ‘PO’: Jaiswal et al., 2005; Planteome Project [http://www.planteome.org ]: Cooper and Jaiswal, 2016; Flora Phenotype Ontology: Hoendorf et al., 2016) so that phenotypic data sets can be incorporated in analyses in a variety of biological fields.

## APPENDIX 2 List of characters and corresponding character states extracted from taxonomic descriptions of the Araucariaceae using natural language processing are contrasted with the characters used in the phylogenetic analysis of Araucariaceae (Escapa and Catalano, 2013).^a^^,^ b

**Table:**

Structures or entities	Natural language processing pipeline^c^	Phenotypic data set (Escapa and Catalano, 2013)^d^
Whole organism	1. Reproduction of organism* (39) 0: monoecious 1: dioecious	Habit0: monoecious1: dioecious
	2. Presence of sap when punctured* (19) 0: milky sap	
Bud	3. Prominence of bud* (38) 0: conspicuous 1: inconspicuous	
Bark	4. Presence of cushion‐shaped scars after branches fall* (39) 0: yes 1: no	
	5. Presence of spongy nodules on bark* (39) 0: yes 1: no	
	6. Coating of bark (4) 0: resinous	
	7. Pubescence_or_Relief of bark (7) 0: rough 1: smooth	
	8. Architecture_or_Pubescence of bark (12) 0: scaly (coarsely scaly, finely scaly, thinly scaly) 1: flaky (coarsely flaky_slightly flaky)	
	9. Condition of bark (25) 0: exfoliating	
	10. Type of exfoliation of bark (21) 0: in large thick flakes 1: in plates 2: in patches 3: in scales (irregular scales, in fine scales) 4: in strips (in thin strips) 5: in circular bands	
	11. Coloration of bark (36) 0: brown (dark‐brown, externally dark‐brown, gray‐brown, grey‐brown, light‐ brown, orangebrown, purplish grey‐brown, purplish‐brown, redbrown) 1: grey (ash‐grey, blue‐grey, gray, light gray, red‐gray) 2: black (light‐brown, purplish‐black) 3: white (externally gray‐white, nearly white, white, whitish) 4: red 5: tan 6: green	
	12. Coloration of inner bark (6) 0: red (internally reddish_reddish) 1: brown (internally reddish‐brown, redbrown) 2: tan 3: pink	
Branching	13. Branching pattern (4) 0: u‐like 1: v‐like	
Resin	14. Coloration of resin (4) 0: white 1: yellow (pale‐yellow_yellowish)	
Branch	15. Orientation of branch (14) 0: horizontal (irregularly horizontal) 1: ascending 2: spreading 3: pendent	
Branchlet	16. Diameter of branchlet (9) 0: 0.0–10.0 1: 10.0–20.0 2: 20.0–30.0 3: 30.0–55.0	
Bud	17. Shape of bud (6) 0: globular 1: round (rounded with scales)	
Leaf	18. Reflectance of leaf (4) 0: glossy (below shiny, shiny) 1: dull	
	19. Texture of leaf (6) 0: coriaceous	
	20. Orientation of leaf (10) 0: spreading 1: incurved (inward)	
	21. Patterns of abaxial side of leaf (12) 0: glaucous (below glaucous, slightly glaucous) 1: non‐glaucous (below non‐glaucous, underneath non‐glaucous)	
	22. Coloration of leaf (16) 0: dark green 1: bright‐green (light green, light‐green, pale‐yellow‐green, yellowish‐green)	
	23. Leaf arrangement* (39) 0: alternate 1: opposite_subopposite 2: spirally (spirally_arranged) 3: tetrastichous	Phyllotaxis of mature leaves0: Helical1: Whorl2: Opposite to subopposite
	24. Arrangement of leaf 2 (14) 0: imbricate (closely imbricate) 1: loosely imbricate	
	25. Shape of leaf 1 (34) 0: lanceolate (narrowly lanceolate, oblong‐lanceolate, oval‐lanceolate, ovate‐lanceolate) 1: elliptic (linear‐elliptic, long‐oval, oblong‐elliptic, oval, ovate‐elliptic) 2: lanceolate 3: circular (round, ovate‐round) 4: ovate (round broadly ovate, triangular‐ovate) 5: lenticular 6: obovate (elliptic‐obovate) 7: triangular	
	26. Shape of leaf 2 (34) 0: laminar blade 1: needlelike 2: scale‐like	
	27. Shape of leaf 3 (14) 0: keeled (dorsally keeled) 1: flattened (somewhat flattened) 2: non‐flattened 3: awl‐shaped	
	28. Shape of leaf apex (21) 0: acute (bluntly acute, sharply acute) 1: obtuse 2: attenuate (acuminate‐attenuate)	Bract/scale fusion at ovuliferous cone0: acute1: obtuse
	29. Length of mature leaf (cm) (8) 0: 0.0–2.5 1: 2.5–5.0 2: 5.0–10.0 3: 10.0–20.0	Mature leaf length (continuous)
	30. Length of juvenile leaf (cm) (8) 0: 0.0–2.5 1: 2.5–5.0 2: 5.0–10.0 3: 10.0–20.0 4: 20.0–25.0	
	31. Width of mature leaf (cm) (5) 0: 0.0–1.0 1: 1.0–5.0 2: 5.0–15.0 3: 15.0–20.0	Mature leaf width (continuous)
	32. Width of juvenile leaf (cm) (16) 0: 0.0–2.0 1: 2.0–4.0 2: 4.0–6.0 3: 6.0–15.0	
Male cone	33. Coloration of male cone (6) 0: brown (redbrown, reddish‐brown, ultimately becoming dark‐brown, yellowish‐brown) 1: bluish‐white 2: reddish	
	34. Architecture_or_Arrangement_or_Growth_Form of male cone (6) 0: solitary 1: in groups	
	35. Position of male cone (39) 0: axillary 1: terminal	Pollen cone disposition0: axillary1: terminal
	36. Fragility_or_Size of peduncle male cone (6) 0: robust (stout)	
	37. Architecture of peduncle of male cone (12) 0: sessile (almost sessile, short peduncle, shortly pedunculate) 1: peduncle (on peduncle)	
	38. Length of male cone (cm) (38) 0: 0.0–5.0 1: 5.0–10.0 2: 10.0–15.0 3: 15.0–26.0	Pollen cone length (continuous)
	39. Shape of male cone (30) 0: cylindrical (broadly cylindrical, cylindric, oblong‐cylindric, ovoid‐cylindrical) 1: globose (globular) 2: pyriform 3: ovate	Pollen cone morphology0: spherical/globose1: ellipsoidal/subglobose2: cylindrical3: irregular
	40. Width of male cone (cm) (35) 0: 0.0–1.0 1: 1.0–2.5 2: 2.5–5.0 3: 5.0–15.0	Pollen cone width (continuous)
Microsporophyll	41. Arrangement of microsporophyll (7) 0: imbricate (strongly imbricate) 1: spirally	Microsporophyll phyllotaxy0: decussate1: helical2: whorled
	42. Shape of microsporophyll (13) 0: triangular (broadly triangular) 1: rhombic 2: oval 3: semicircular	
	43. Shape of microsporophyll apex (4) 0: umbonate 1: acute 2: obtuse	
Midrib	44. Prominence of midrib (13) 0: prominent (visible) 1: faint (not conspicous)	Midrib0: evident from external view1: not evident from external view
Female cone	45. Coloration of female cone (9) 0: green (glaucous‐green, greenish, olive‐green) 1: brown (purplish brown, chestnut‐brown, dark‐brown, when ripe brown)	
	46. Length of female cone (cm) (29) 0: 0.0–10.0 1: 10.0–20.0 2: 20.0–35.0	Ovuliferous cone length (continuous)
	47. Width of female cone (cm) (29) 0: 0.0–10.0 1: 10.0–20.0 2: 20.0–25.0	Ovuliferous cone width (continuous)
	48. Shape of female cone (39) 0: globose (globular, subglobose) 1: elliptic (broadly ellipsoidal, globose‐ovoid, oval, ovoid) 2: obovate 3: lanceolate	Ovuliferous cone morphology0: spherical/globose1: ellipsoidal/subglobose2: cylindrical3: irregular
	49. Fusion of bracts and scales of female cone (4) 0: yes	Bract/scale fusion at ovuliferous cone0: absent1: present
	50. Length of female cone scale (cm) (4) 0: 0.0–3.0 1: 3.0–4.0	
Ovate scales	51. Shape of ovate scales (39) 0: flattened (somewhat flattened)	
	52. Shape of ovate scales 2 (39) 0: broadly ovate 1: thin	
Scales	53. Arrangement of scale (7) 0: imbricate 1: densely imbricate	
	54. Shape of scale (18) 0: round (broadly rounded) 1: triangular (nearly triangular, roughly triangular) 2: angular 3: ovate (ovoid) 4: lanceolate 5: quadrangular	
	55. Seed cone scales apical appendage (18) 0: yes	
	56. Shape of apex of scale (5) 0: well rounded 1: obtuse 2: acuminate	
Cone bract	57. Shape of cone bract (4) 0: oblong‐elliptic 1: oblong‐ovate 2: acuminate 3: triangular	
	58. Length of cone bract (mm) (12) 0: 0.0–10.0 1: 10.0‐–20.0	
Bract	59. Orientation of bract (9) 0: recurved 1: erect 2: incurved 3: reflexed	
Nut	60. Size_or_Width of nut (5) 0: broad 1: narrow (relatively narrow)	
	61. Shape of nut (7) 0: oblong 1: ovate 2: triangular 3: somewhat rectangular	
Scale	62. Width of scale (cm) (5) 0: 0.0–3.0 1: 3.0–6.0	
Seeds	63. Seeds becoming detached 0: yes 1: no scales* (39)	Seed abscission0: absent1: present
	64. Shape of seed (12) 0: ovoid (ellipsoid_oval, oblong‐subovoid) 1: obovoid 2: cordate (narrowly cordate) 3: rounded 4: triangular	
	65. Width of seed (mm) (12) 0: 0.0–20.0 1: 20.0–50.0 2: 50.0–90.0	Seed width (continuous)
	66. Length of seed (cm) (26) 0: 0.0–1.0 1: 1.0–4.0 2: 4.0–10.0 3: 10.0–20.0	Seed length (continuous)
	67. Wings on seeds (39) 0: protruding wing on one side and small protrusion on the other 1: wingless 2: two wings 3: circumferentially winged	Integumentary seed wings0: absent1: presentIntegumentary seed wing symmetry0: 11: 2Integumentary seed wing symmetry0: asymmetric1: symmetric
	68. Length of wing of seed (mm) (5) 0: 0.0–10.0 1: 10.0–20.0 2: 20.0–30.0	
	69. Shape of wing of seed (11) 0: truncated 1: obovoid 2: rounded (broadly rounded) 3: ovate 4: ovate (broadly ovate) 5: triangular 6: rectangular	
Cotyledons	70. Quantity of cotyledon (15) 0: 2 1: 4	Number of cotyledons0: 21: 42: cotyledon tube
	71. Germination of cotyledon (19) 0: epigeal (reportedly epigeal) 1: hypogeal	Germination0: epigeal1: cryptogeal

^*^Characters extracted from generic descriptions that were manually added to the matrix.

a Expressions included in parentheses were coded under the same character state because they were considered synonymous.

b Terms or expressions are presented in the format that they were extracted by the pipeline from the source literature. Underscore (e.g., “ellipsoid_oval”) signifies “to” or “or” (i.e., “ellipsoid to oval” or “ellipsoid or oval”).

c Character name (no. of taxa with data); code: character state.

d Equivalent character(s); code: character state.
