# Supplementary material for: Extraction of phenotypic traits from taxonomic descriptions for the tree of life using natural language processing
Source: Appl Plant Sci. 2018 Mar 31;6(3):e1035. doi: 10.1002/aps3.1035 (PMC5895189; doi:10.1002/aps3.1035)
Supplement: Supplementary file 1 [file APS3-6-e1035-s001.docx]

APPENDIX S1. Final phenotypic matrix of the Araucariaceae. Characters and traits were extracted from taxonomic descriptions using a natural language processing pipeline and discretized by the user. Characters and character states can be interpreted using Appendix 2.

Character 1 2 3 4 5 6 7 8 9 10 11 12 13 14 15 16 17 18 19 20 21 22 23 24 25 26 27 28 29 30 31 32 33 34 35 36 37 38 39 40 41 42 43 44 45 46 47 48 49 50 51 52 53 54 55 56 57 58 59 60 61 62 63 64 65 66 67 68 69 70 71

Agathis atropurpurea 0 0 0 0 1 ? ? 0 ? ? 0,2 ? ? ? ? ? 0 ? ? ? 0 ? 2,1,0 0 0,1 ? ? ? 1 ? 0,1 ? ? ? 0 ? ? 0 ? 0 0 ? ? ? 0 ? 0 0 ? ? 0 0 ? ? ? ? ? ? ? ? ? ? 0 ? ? 0,1 0 ? ? ? ?

Agathis australis 0 0 0 0 1 ? ? ? 0 0 1 ? ? ? 0 ? 1 ? 0 ? 0 0 0,1 ? 2,0 ? ? ? 0,1 2,3 0,1 0 ? ? 0 0 0 0,1 0 ? ? ? ? ? 0 ? 0 0 ? ? 0 0 0 ? ? ? ? ? ? ? ? ? 0 ? ? ? 0 ? ? ? 0,1

Agathis borneensis 0 0 0 0 1 ? 0 0 0 ? 1,2 ? ? ? ? ? ? ? 0 ? ? 1 1 ? ? ? ? 0 1,3 ? ? ? 0 0 0 0 ? 0 0 1 ? ? ? ? ? ? ? 1 ? ? 0 0 ? ? ? ? ? ? ? ? ? ? 0 ? ? ? 0 ? 0 ? ?

Agathis corbassonii 0 0 0 0 1 0 ? 0 ? ? 5,0 ? ? ? ? ? ? ? ? ? 0 ? 2,1,0 ? 2,1 0 ? 1 1,2 3 0,1 0,1 ? ? 0 ? ? 0,1 0 0,1 0 ? ? ? ? 0,1 ? 2 ? ? 0 0 ? 0 ? ? ? ? ? ? 0 ? 0 0 0 0,1 0 ? ? ? ?

Agathis dammara 0 0 0 0 1 ? 0 ? 0 ? 1,0,2 ? ? ? ? ? ? ? 0 ? ? 1,0 1 ? 2,1 0 ? 2 1,2 1,2 1 1,3 ? 0 0 0 1 0 ? 0 0 ? ? ? ? 0,1 0 0,1 ? ? 0 0 ? 1 ? ? ? ? ? ? ? ? 0 0 ? 0,1 0 0,1 ? ? ?

Agathis endertii 0 0 0 0 1 ? ? ? ? ? 0 ? ? ? ? ? ? ? ? ? 0 ? 2,1,0 ? 1 0 ? 0 2 ? 1 ? ? ? 0 ? ? 0 ? 1,2 ? ? ? ? ? 0 ? 1 ? ? 0 0 ? 1 ? 0 ? ? ? ? ? ? 0 1 0 1 0 ? ? ? ?

Agathis flavescens 0 0 0 0 1 ? ? ? ? ? 6,0 ? ? ? ? ? ? ? ? ? 0 1 2,1,0 ? 6 0 ? 1 1,2 ? 1 ? ? ? 0 ? ? 0 ? 1 ? ? ? ? 1 0 0 0 ? ? 0 0 ? ? ? ? ? ? ? ? ? 0 0 0 0 1 0 0,1 ? ? ?

Agathis kinabaluensis 0 0 0 0 1 ? 1 ? 0 1 0 0 ? 0 ? ? ? ? ? ? 1 ? 2,1,0 ? 3 0 ? 2,1 1,2 ? 1 ? ? ? 0 ? 0 0 0 0 ? ? ? ? ? 0,1 0 1 ? ? 0 0 ? 1 ? ? ? ? ? ? ? ? 0 ? ? ? 0 ? ? ? ?

Agathis labillardieri 0 0 0 0 1 ? ? ? ? ? 0 ? ? ? ? ? ? 0 ? ? ? ? 2,1,0 ? 0,1 0 ? ? 2,3 ? 1 ? ? ? 0 0 0 0 0 1 ? ? 0 ? 0 0 0 0 ? ? 0 0 0 2 ? ? ? ? ? ? ? ? 0 ? 0 1 0 ? ? ? ?

Agathis lanceolata 0 0 0 0 1 ? ? 0 ? ? 0 ? ? ? 1 ? 0 ? ? ? 1 0 2,1,0 ? 2,1 0 ? 3 2 2 1 0,1 ? ? 0 ? 0 0 0 0,1 0 ? ? ? ? 1 1 1,0 ? ? 0 0 0 0 ? ? ? ? ? ? ? ? 0 0 0 1 0 2 ? ? ?

Agathis lenticula 0 0 0 0 1 ? ? 1 0 1 3 1 ? 0 ? ? ? ? ? ? ? ? 2,1,0 ? 3,5 0 ? 2 2 0,3 1 2 ? ? 0 ? 0 0 0 1 ? ? ? ? ? 0 0 1 ? 0 0 0 ? ? ? ? ? ? ? ? ? 1 0 ? ? ? 0 ? ? ? ?

Agathis macrophylla 0 0 0 0 1 ? ? 0 0 2 1 0 ? ? 2,1 ? ? ? 0 ? 0 1 1 ? ? 0 ? ? 1,2 2,3 1,2 0,1 ? 0 0 0 ? 0 ? 0,1 0 ? ? ? 0,2 ? 1 0 ? 1 0 0 ? ? ? ? ? ? ? ? ? 1 0 0 0 1 0 2 ? ? ?

Agathis microstachya 0 0 0 0 1 ? ? 1 ? ? 0 3 ? ? ? ? ? ? ? ? ? ? 1 ? 1 0 ? ? 0,2 2 0,1 0,1 ? ? 0 ? 0 0 ? 0 ? ? ? ? ? 0,1 0,1 0,1 ? 0,1 0 0 ? ? ? ? ? ? ? ? ? 1 0 2 ? ? 0 ? ? 0 ?

Agathis montana 0 0 0 0 1 ? ? ? 0 ? 5 1 ? ? ? ? 0 ? ? ? ? ? 2,1,0 ? 0 0 ? 3 2 2,3 1 1 ? ? 0 ? ? 0,1 1 0,1 ? ? ? ? ? ? ? 1 ? ? 0 0 0 3 ? 1 ? ? ? ? ? ? 0 3 ? ? 0 ? 1 ? ?

Agathis moorei 0 0 0 0 1 ? ? ? 0 3 3 2,0 ? ? 3 ? 1 ? ? ? 0 0 1 ? 2,1 0 ? 3 2 4 0,1 1 ? ? 0 ? ? 2,3 1,2 3 ? ? ? ? ? ? ? 1 ? ? 0 0 0 0 ? ? ? ? ? ? ? ? 0 ? ? ? 0 ? ? 0 ?

Agathis orbicula 0 0 0 0 1 ? ? ? 0 1 0 ? ? 1 ? ? ? ? ? ? 0 ? 2,1,0 0 3 0 ? 0,3 0,1 2 1 0,1 ? ? 0 ? 0 1,2 3,0 2,3 ? ? ? ? ? ? ? 1 ? ? 0 0 ? ? ? ? ? ? ? ? ? ? 0 ? ? ? 0 ? ? ? ?

Agathis ovata 0 0 0 0 1 ? ? ? ? ? 3,5 1 ? ? ? ? 1 ? ? ? 0 0,1 2,1,0 ? 4,1 0 ? ? 1,2 ? 1,2 ? ? ? 0 0 0 0,1 0 1 0 0 ? ? ? 0,1 1 0 ? ? 0 0 1 ? ? ? ? ? ? 0 ? ? 0 ? 0 0,1 0 ? ? ? ?

Agathis robusta 0 0 0 0 1 ? 1 1 ? ? 0 0,3,1 ? ? ? ? ? 0,1 ? ? ? 0 2,1 ? 0,1 0 ? 0 2,3 2 1 0,1 ? ? 0 ? 0 0,1 0 0 ? ? ? ? ? ? ? 1 ? ? 0 0 ? ? ? ? ? ? ? ? ? ? 0 2 ? ? 0 ? ? 0 ?

Agathis silbae 0 0 0 0 1 ? 1 0 0 ? 1,3 0,3 ? 0,1 2,1 ? ? ? 0 ? ? 1 1 ? ? 0 ? ? 0,2 0,1 1,2 ? 0 0 0 ? 1 0 1 2,3 ? ? ? ? 0,2 0 1 0 ? 1 0 0 ? 1 ? ? ? ? ? ? ? 1 0 0 2 3 0 2 ? ? ?

Araucaria angustifolia 1 ? 1 1 1 0 ? 0 ? ? ? ? ? ? 0 ? ? ? ? ? ? ? 2 ? 2 1 ? 0 1,2 ? 0 ? ? ? 0,1 ? ? 2,3 0 1,2 ? ? ? 0 2 1,2 1 0 ? ? 1 1 0 ? 0 ? ? ? 0 ? ? ? 1 ? 0,1 2 1,2 ? ? 0 1

Araucaria araucana 1,0 ? 1 1 1 0 1 ? ? ? 0 ? ? ? 0 ? ? 0 ? ? ? 0 2 ? 0 2 ? ? 1,2 ? 0,1 ? 0 0,1 0,1 ? ? 1,3 ? 3 ? ? 1 ? 2 1 0,1 0 ? ? 1 1 ? ? 0 ? ? ? ? 1 ? ? 1 4 0 1,2 2 ? ? ? ?

Araucaria bernieri 1 ? 1 1 1 ? ? ? 0 4 1 ? 0 ? ? 0 ? ? ? ? ? ? 2 0 7 1,2 0 ? 0 ? 0 ? 1 ? 0,1 ? ? 0 0 0,1 ? 0 ? ? ? 0,1 0 0 ? ? 1 1 ? 4 0 ? ? 0 ? ? 1 ? 1 ? ? 0,1 2 ? 2 0 0

Araucaria bidwillii 0 ? 1 1 1 ? ? ? ? ? 0,2 ? ? ? ? ? ? 0 0 0 ? 1,0 2 ? 2,4 ? 1 ? 0,1 ? 1,2 ? ? 0 0 ? ? 3 0 ? ? ? ? ? ? 2 2 0,1 ? ? 1 1 ? ? 0 ? 0,1 ? ? ? ? ? 1 ? ? ? 1,2 ? ? ? ?

Araucaria biramulata 1 ? 1 1 1 ? ? ? 0 4 1,0 ? ? ? 2 ? ? ? ? 0 ? ? 2 ? 4 2 ? 2 0,2 ? 0 ? ? ? 0,1 ? ? 1 0 1 ? 0 ? 0 ? 0,1 0 0 ? ? 1 1 ? ? 0 2 ? 0 1 ? ? ? 1 ? ? 0,1 2 ? 3 ? 0

Araucaria columnaris 1 ? 1 1 1 ? ? ? 0 4 1 ? ? ? ? ? ? ? ? ? ? ? 2 0 2,7 1,2 ? 2,1 0 0 0 0 ? ? 0,1 ? ? 1 0 1 ? ? ? 1 ? 1 0,1 0 ? ? 1 1 ? 1 0 ? ? 0 ? ? 1 ? 1 ? ? 1 2 ? 2 ? 0

Araucaria cunninghamii 0 ? 1 1 1 ? 0 ? 0 5 ? ? ? ? ? 0,2 ? ? ? 1 0 0 2 0 ? 2 0 0 0 ? ? ? ? ? 0,1 ? ? 0 0 0 ? 1 2 ? ? 0,1 0 1 ? ? 1 1 ? ? 0 ? ? ? ? ? ? ? 1 ? 0 1 1,2 ? ? 0 0

Araucaria heterophylla 1 ? 1 1 1 ? ? ? 0 3 0 ? ? ? 0,3 ? ? ? ? 1 ? 0 2 0 4 1 3 ? 0 0 0 ? 0,2 ? 0,1 ? ? 0 ? ? ? ? 1 ? ? 1 ? 0 ? ? 1 1 ? 1 0 ? ? ? 2 ? ? ? 1 ? 0 1 1,2 ? ? 1 0

Araucaria humboldtensis 1 ? 1 1 1 ? ? ? 0 4 1 ? 1 ? ? ? ? ? ? ? ? ? 2 ? 7,4 2 0 ? 0 0 0 0 ? ? 0,1 ? ? 1 0 1 ? 0 ? 0 ? 0 0 0 ? ? 1 1 ? 5 0 ? ? 0 1 ? ? ? 1 ? ? 1 1,2 ? ? 0 0

Araucaria hunsteinii 1 ? 1 1 1 0 ? ? 0 1 0,4 ? ? ? ? ? ? ? ? ? ? ? 2 ? 2 1,2 1 3 2,3 ? 1 ? ? ? 0,1 ? ? 3 0 1,2 ? ? ? ? ? 1,2 1 3 ? ? 1 1 ? ? 0 ? ? ? 0 1 ? ? 1 4 ? ? 1,2 ? ? 0 0

Araucaria laubenfelsii 1 ? 1 1 1 ? ? ? 0 4,3 1 ? ? ? 2 1,2 ? ? ? 0 ? ? 2 0 7 1,2 2 ? 0 ? 2 ? ? ? 0,1 ? ? 2,3 0 1,2 ? 0 ? 0 ? 1 0 0 ? ? 1 1 ? 3 0 ? ? 0,1 1 0 ? ? 1 ? ? 1 2 ? 2 1 0

Araucaria luxurians 1 ? 1 1 1 ? ? ? 0 4 1 ? ? ? ? 1 ? ? ? 0 ? ? 2 0 4 2 2 ? 0 ? 0 ? ? ? 0,1 ? ? 2,3 0 2 ? 2 ? 0 ? 1 0,1 0 ? ? 1 1 ? 1 0 2 ? 0,1 ? ? ? ? 1 ? ? 1 2 ? 4 0 0

Araucaria montana 1 ? 1 1 1 ? ? 0 0 4 0,1 ? ? ? 2 ? ? ? ? ? ? ? 2 1 4,2 2 2 0,1 0 0 1 0 ? ? 0,1 ? ? 1,2 0 1,2 ? 0 ? 0 ? 0 0 0 ? ? 1 1 ? 1 0 ? 2 0,1 ? ? ? ? 1 ? ? 1 2 ? 3 1 0

Araucaria muelleri 1 ? 1 1 1 ? ? ? 0 4 1 ? ? ? 2 3 ? ? ? ? ? ? 2 0 7 2 1 0 1 0,1 1 ? ? ? 0,1 ? ? 2,3 0 2 ? ? ? 0 ? 1 0,1 0 ? ? 1 1 ? ? 0 2 ? 1 1 1 2 ? 1 ? ? 1 1,2 ? ? ? 0

Araucaria nemorosa 1 ? 1 1 1 ? ? ? ? ? ? ? ? ? ? 0,1 ? ? ? 1 ? ? 2 0 2 1,2 0 ? 0 ? 0 ? ? ? 0,1 ? ? 1 0 1 ? 0 ? 0 ? 1 0 1 ? ? 1 1 ? ? 0 ? ? 1 3 ? 3 ? 1 ? ? 0 2 ? 3 1 0

Araucaria rulei 1 ? 1 1 1 ? ? 0 0 4 0 ? ? ? ? 2 ? ? ? 1 ? 0 2 0 2 2 ? 0 0,1 ? 0 ? ? ? 0,1 ? ? 2 0 2 ? 0 ? 0 ? 1 0 0 ? ? 1 1 ? 1 0 ? 3 0,1 1 ? ? ? 1 ? ? 0,1 2 ? 3 ? 0

Araucaria schmidii 1 ? 1 1 1 ? ? ? 0 4 1 ? 0 ? 2 0 ? ? ? ? ? ? 2 0 2 1 0 ? 0 0 0 0 ? ? 0,1 ? ? ? ? ? ? ? ? ? ? ? ? 0 ? ? 1 1 ? ? 0 ? ? ? ? ? ? ? 1 ? ? ? 1,2 ? ? 1 0

Araucaria scopulorum 1 ? 1 1 1 ? ? ? 0 4 1,3 ? 0 ? 2 0 ? ? ? 1 ? ? 2 0 4 1,2 ? ? 0 0 0 ? ? ? 0,1 ? ? 0,1 0 0,1 ? 0 ? 0 ? ? ? 0 ? ? 1 1 ? 3 0 ? 3 0 ? ? 0 ? 1 ? ? 0,1 2 ? ? ? 0

Araucaria subulata 1 ? 1 1 1 ? ? ? 0 4 1 ? ? ? ? ? ? ? ? 1 ? ? 2 ? 0 1,2 0 ? 0 ? 0 ? ? ? 0,1 ? ? 1,2 0 1 ? 0 ? 0 ? 1 0 0 ? ? 1 1 ? ? ? ? ? 0 ? ? 1 ? 1 ? ? 0,1 2 ? 5 ? 0

Wollemia nobilis 0 ? ? 1 0 ? ? ? ? ? 0 ? ? ? ? ? ? ? ? ? ? 1 3 ? ? ? 0 0 0 0 0 0 0 ? 1 ? 0 1,2 0 1 1 0,3 ? 1 0 0,1 0,1 0,1 0 ? 1 1 ? ? ? ? ? ? ? ? ? ? 1 ? ? ? 3 ? ? 0 0
